# Supplementary material for: Host Species and Environmental Effects on Bacterial Communities Associated with Drosophila in the Laboratory and in the Natural Environment
Source: PLoS One. 2013 Aug 13;8(8):e70749. doi: 10.1371/journal.pone.0070749 (PMC3742674; doi:10.1371/journal.pone.0070749)
Supplement: Figure S1 — P-value distributions for ANOVAs testing the alternative hypothesis that microbial communities differ between wild caught D. melanogaster and D. simulans based on PCoA of Jaccard distances. If there was no species effect on microbial community composition, p-values are expected to be uniformly distributed. PCos 1–9 are displayed. Axes 3, 4, and 5 are enriched for low p-values indicating a species effect. (PDF) [file pone.0070749.s001.pdf]

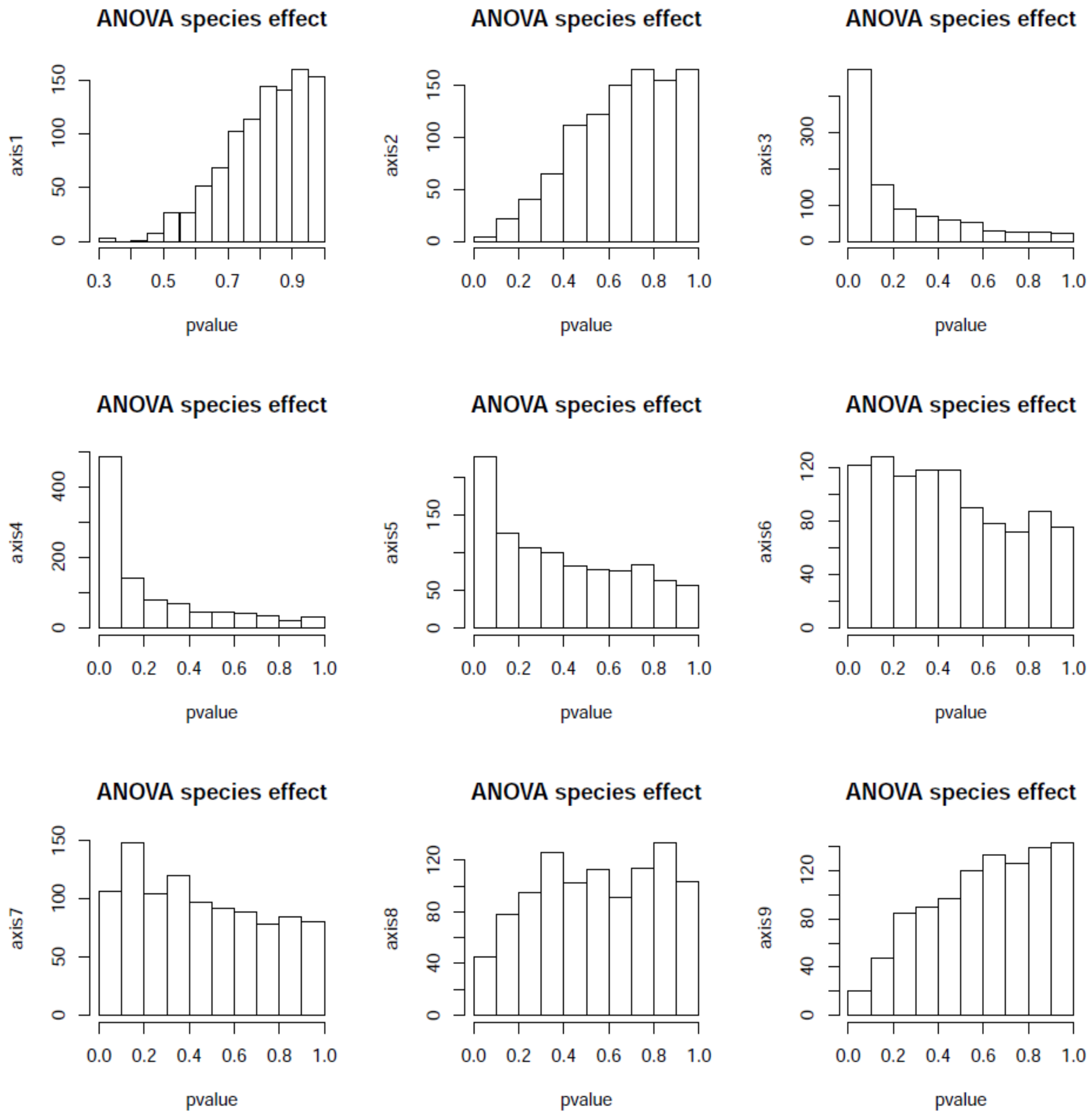

Figure S1
P-value distributions for ANOVAs testing the alternative hypothesis that microbial communities differ between wild caught *D. melanogaster* and *D. simulans* based on PCoA of Jaccard distances. If there was no species effect on microbial community composition p-values are expected to be uniformly distributed. PCos 1-9 are displayed. Axes 3, 4, and 5 are enriched for low p-values indicating a species effect.
