# Supplementary material for: Host Species and Environmental Effects on Bacterial Communities Associated with Drosophila in the Laboratory and in the Natural Environment
Source: PLoS One. 2013 Aug 13;8(8):e70749. doi: 10.1371/journal.pone.0070749 (PMC3742674; doi:10.1371/journal.pone.0070749)
Supplement: Table S2 — Non-significance of a correlation between bacterial community distances and genetic distances for the nine lab-reared Drosophila species obtained from the UCSD Stock Center. The genetic distances between the nine Drosophila species were calculated genome wide using 4-fold degenerate sites. We tested for correlation of the genetic distances with different community distance measures at different OTU identity cutoffs. P-values based on Pearson's correlation coefficient and Spearman's rho are shown. (PDF) [file pone.0070749.s003.pdf]

Distance method	OTU threshold	Pearson p-value	Spearman p-value
Jaccard	97%	0.69	0.63
Jaccard	95%	0.57	0.97
Jaccard	90%	0.64	0.61
Jaccard	80%	0.79	0.34
Jaccard	unique	0.39	0.50
Bray-Curtis	97%	0.70	0.30
Bray-Curtis	95%	0.83	0.37
Bray-Curtis	90%	0.83	0.39
Bray-Curtis	80%	0.97	0.24
Bray-Curtis	unique	0.09	0.17
Morisita-Horn	97%	0.66	0.30
Morisita-Horn	95%	0.64	0.39
Morisita-Horn	90%	0.64	0.39
Morisita-Horn	80%	0.79	0.26
Morisita-Horn	unique	0.07	0.23
ThetaYC	97%	0.77	0.30
ThetaYC	95%	0.64	0.39
ThetaYC	90%	0.64	0.39
ThetaYC	80%	0.81	0.26
ThetaYC	unique	0.07	0.23
Unifrac	na	0.45	0.15
Weighted Unifrac	na	0.33	0.15

Supplementary table 2
Non-significance of a correlation between bacterial community distances and genetic distances for the nine lab-reared *Drosophila* species obtained from the UCSD Stock Center. The genetic distances between the nine *Drosophila* species were calculated genome wide using 4-fold degenerate sites. We tested for correlation of the genetic distances with different community distance measures at different OTU identity cutoffs. P-values based on Pearson's correlation coefficient and Spearman's rho are shown.
